# *De novo* gene integration into regulatory networks via interaction with conserved genes in peach

**DOI:** 10.1093/hr/uhae252

**Published:** 2024-09-05

**Authors:** Yunpeng Cao, Jiayi Hong, Yun Zhao, Xiaoxu Li, Xiaofeng Feng, Han Wang, Lin Zhang, Mengfei Lin, Yongping Cai, Yuepeng Han

**Affiliations:** State Key Laboratory of Plant Diversity and Specialty Crops, Wuhan Botanical Garden, Chinese Academy of Sciences, Wuhan 430074, China; College of Life Sciences, Anhui Agricultural University, Hefei 230036, China; State Key Laboratory of Plant Diversity and Specialty Crops, Wuhan Botanical Garden, Chinese Academy of Sciences, Wuhan 430074, China; Beijing Life Science Academy, Beijing 102209, China; College of Life Sciences, Anhui Agricultural University, Hefei 230036, China; Key Laboratory of Horticultural Crop Germplasm Innovation and Utilization (Co-construction by Ministry and Province), Institute of Horticulture, Anhui Academy of Agricultural Sciences, Hefei 230000, China; Hubei Shizhen Laboratory, Hubei Key Laboratory of Theory and Application Research of Liver and Kidney in Traditional Chinese Medicine, School of Basic Medical Sciences, Hubei University of Chinese Medicine, Wuhan 430065, China; Jiangxi Provincial Key Laboratory of Plantation and High Valued Utilization of Specialty Fruit Tree and Tea, Institute of Biological Resources, Jiangxi Academy of Sciences, Nanchang 330224 Jiangxi, China; College of Life Sciences, Anhui Agricultural University, Hefei 230036, China; State Key Laboratory of Plant Diversity and Specialty Crops, Wuhan Botanical Garden, Chinese Academy of Sciences, Wuhan 430074, China

## Abstract

*De novo* genes can evolve “from scratch” from noncoding sequences, acquiring novel functions in organisms and integrating into regulatory networks during evolution to drive innovations in important phenotypes and traits. However, identifying *de novo* genes is challenging, as it requires high-quality genomes from closely related species. According to the comparison with nine closely related *Prunus* genomes, we determined at least 178 *de novo* genes in *P. persica* “baifeng”. The distinct differences were observed between *de novo* and conserved genes in gene characteristics and expression patterns. Gene ontology enrichment analysis suggested that Type I *de novo* genes originated from sequences related to plastid modification functions, while Type II genes were inferred to have derived from sequences related to reproductive functions. Finally, transcriptome sequencing across different tissues and developmental stages suggested that *de novo* genes have been evolutionarily recruited into existing regulatory networks, playing important roles in plant growth and development, which was also supported by WGCNA analysis and quantitative trait loci data. This study lays the groundwork for future research on the origins and functions of genes in *Prunus* and related taxa.

## Introduction

The origin of new genes with novel functions drives adaptive evolution and the innovation of lineage-specific phenotypes [1, 2]. New genes can emerge through various processes, such as horizontal gene transfer, retrotransposition, gene fission, gene fusion, gene duplication, and *de novo* origin [3–7]. Horizontal gene transfer moves genetic material between species, aiding rapid adaptation. Retrotransposition involves RNA being reverse-transcribed into DNA and reinserted into the genome, potentially forming new gene copies. Gene fission and fusion alter gene structure by splitting or merging genes, resulting in novel functionalities. Gene duplication produces additional gene copies that may evolve new functions. *De novo* gene origination involves the transformation of previously noncoding DNA sequences into functional genes, a key mechanism underpinning phenotypic innovation and structural variation [5, 7–13]. These genes, which have gained significant attention in recent years, are recognized as major contributors to genetic innovation [5, 7–15]. *De novo* genes typically evolve rapidly, exhibiting distinct features such as highly diversified sequences, lower expression levels, and shorter lengths compared to conserved genes. The process of *de novo* gene birth generally involves two key steps: first, open reading frames (ORFs) are generated from noncoding sequences through mechanisms like exonization or overprinting; second, these ORFs are translated or transcribed into proteins or mRNAs [9, 16–18]. This integration into regulatory networks allows *de novo* genes to drive important evolutionary changes and adaptations. The identification of *de novo* genes requires two essential criteria: (i) the gene must demonstrate translation or transcriptional activity and (ii) the availability of high-quality genomes for both the focal species and its close relatives [9, 16–19]. For example, according to the comparison with closely related *Arachis* genomes, Song *et al.* [9] determined 381 *de novo* genes in *Arachis hypogaea* cv. Tifrunner.

The first experimental confirmed *de novo* gene was described by Begun *et al.* and Levine *et al.* [20, 21]. Cai *et al.* [22] reported on the function of the *de novo* gene *BSC4* in yeast (*Saccharomyces cerevisiae*), discovering that it plays a role in synthetic lethal interactions involved in DNA repair. Subsequently, more such genes have been identified across various taxa, including *Drosophila*, primates, rodents, fungi, and plants [8, 9, 19, 22–29]. Numerous studies have drawn several key conclusions about *de novo* genes: (i) they are present in nearly all organisms; (ii) *de novo* and conserved genes exhibit distinct genomic characteristics; and (iii) *de novo* genes can acquire specific biological functions [7, 16, 30, 31]. The advent of high-throughput sequencing technology has led to the sequencing of an increasing number of high-quality plant genomes, significantly enhancing our ability to identify *de novo* genes. Subsequently, the systematic identification of *de novo* genes has been conducted in various plants, including species within the subfamily *Bambusoideae*, *Oryza sativa*, bamboo, *Arabidopsis thaliana*, and *A. hypogaea* cv. Tifrunner [9, 16, 32, 33]. Li *et al.* [32] discovered 782 *de novo* genes in *A. thaliana*, underscoring the potential pivotal roles of epigenetic modifications in the emergence of these genes. Zhang *et al.* [16] identified 175 *de novo* genes in *O. sativa* subspecies *japonica* and found that these genes exhibit high diversity in their evolutionary patterns and have undergone rapid evolution. Jin *et al.* [33] observed that *de novo* genes exhibit higher expression levels in bamboo shoots, correlating with their rapid growth. In *A. hypogaea* cv. Tifrunner, Song *et al.* [9] identified 381 *de novo* genes, demonstrating their multifunctional roles in growth, development, and biotic stress responses. In plants, the first experimentally confirmed *de novo* gene is *QQS* in *A. thaliana*, which reduces susceptibility to pathogens and pests while regulating nitrogen and carbon metabolism [34, 35]. In *O. sativa* subspecies *japonica*, Xiao *et al.* [36] found that a *de novo* gene, *OsDR10*, can help plants resist infection by *Xanthomonas oryzae* pv. *Oryzae*. The rice *de novo* gene *GSE9* influences grain shape differences between *japonica/geng* and *indica/xian* varieties [37].

**Figure 1:**
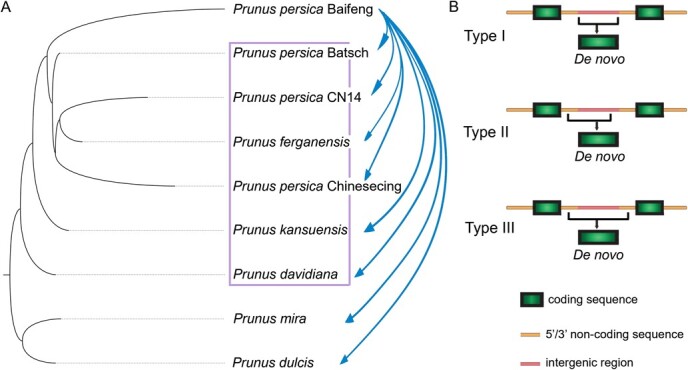
Identification of recently emerged *de novo* genes during diversification in the genus *Prunus*. (A) Through BLAT searches of *P. persica “*baifeng*”* against sibling *Prunus* species, 178 *De novo* genes were determined in *P. persica “*baifeng*”.* Three cultivars, *P. persica* “CN14”, *P. persica* “Batsch” and *P. persica* “Chinese Cling”, and three wild relatives, *P. ferganensis*, *P. davidiana*, and *P. kansuensis*, were referred to as an in-group, while *P. mira* and *P. dulcis* were defined as an out-group. (B) Three types of *de novo* genes are shown in the diagram.

*De novo* genes can generally be categorized into distinct types. Based on selective pressure patterns, *de novo* genes can be classified into three distinct types: Type I, Type II, and Type III [9, 30]. Type I genes are absolutely derived from noncoding sequences [38], while Type II and III genes are constituted of chimeric sequences that contain a minority of transposable elements or pre-existing genes [9, 30]. Additionally, according to the basis of transcription, translation, or an absence of translation and transcription, *de novo* genes from *A. thaliana* can be classified into three types by Li *et al.* [32]. Peach (*Prunus persica*) represents a globally significant perennial fruit crop with substantial economic importance [39, 40]. The release and completion of high-quality genomes for several *Prunus* species [41–45] position them as a model system for the detection of *de novo* genes. Here, we assembled the transcriptomes of different tissues from *P. persica* “baifeng”. By comparing the sequences of *P. persica* “baifeng” with those of closely related *Prunus* genomes, we detected *de novo* genes and analyzed their expression patterns, evolutionary pathways, and functions. Collectively, our findings offer a comprehensive view of *de novo* genes in peach, shedding light on their evolutionary roles and functional implications.

## Results

### Search for *de novo* genes in peach

In our study, we first used all protein sequences of *P. persica* “baifeng” to search against the local protein databases of other closely related *Prunus* plants, including *P. persica* “CN14”, *P. persica* “Batsch”, *P. kansuensis*, *P. persica* “Chinese Cling”, *P. ferganensis*, *P. mira*, *P. dulcis*, and *P. davidiana*. Three cultivars, *P. persica* “CN14”, *P. persica* “Batsch” and *P. persica* “Chinese Cling”, and three wild relatives, *P. ferganensis*, *P. davidiana*, and *P. kansuensis*, were referred to as an in-group, while *P. mira* and *P. dulcis* were defined as an out-group. We only kept these sequences that did not have any orthologues identified in other closely related *Prunus* genomes as candidate genes for further analysis (Fig. 1A). These specific DNA sequences were mapped onto both out-group and in-group genomes and were classified as putative *de novo* genes if they could be mapped to noncoding regions in at least one in-group genome and one out-group genome, as described by Zhang *et al.* [46]. Subsequently, we aligned all *Prunus* sequences with the putative *de novo* genes and excluded one *de novo* gene because it originated from an open ORF in a noncoding region. Finally, we identified 178 specific DNA sequences as candidate *de novo* genes in *P. persica* “baifeng” (Table S1).

**Figure 2:**
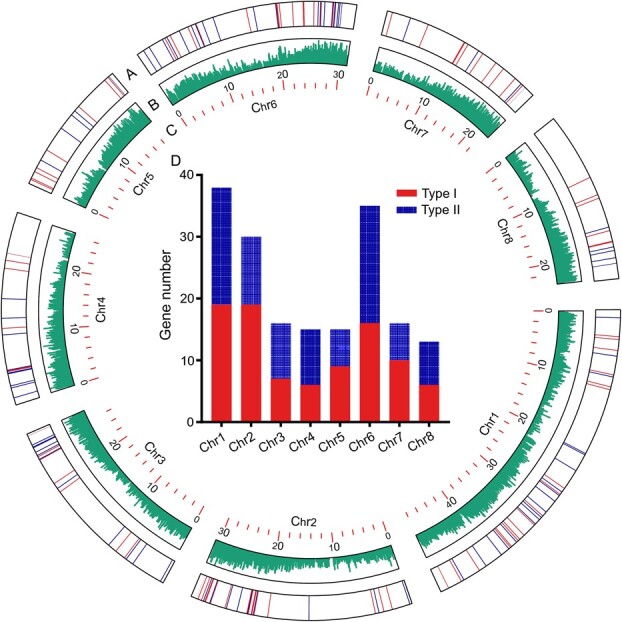
Chromosomal distribution of expressed *de novo* genes. (A) The chromosomal locations of expressed *de novo* genes. (B) The gene density in the genome of *P. persica “*baifeng*”.* (C) The chromosomes of *P. persica “*baifeng*”.* D. The numbers of Type I, and Type II *de novo* genes across *P. persica “*baifeng*”.*

To determine whether the candidate *de novo* genes were transcriptionally active, we performed transcriptome sequencing of different tissues from *P. persica* “baifeng”. Our findings indicate that 158 out of 178 analyzed genes showed expression in at least one type of tissue, thereby confirming their identification as *de novo* genes in *P. persica* “baifeng” (Table S2). According to McLysaght and Hurst [30], *de novo* genes are classified into three types: Type I genes arise entirely from noncoding sequences, Type II genes originate from a small number of pre-existing genes or transposable elements, and Type III genes are composed of chimeric sequences. In our study, 75 Type I *de novo* genes are entirely constituted of ancestral noncoding sequences, 83 Type II genes originate from chimeric sequences incorporating minor portions of pre-existing genes, and no Type III genes are composed entirely of noncoding sequences (Table S2, Figs 1B, and 2).

The peach genome comprises eight chromosomes, Chr 1–Chr 8 [41–45]. To determine the distribution of *de novo* genes on chromosomes, we used the gff3 file to obtain the location information of each gene. The physical locations of the *de novo* genes were unevenly distributed across all *P. persica* “baifeng” chromosomes (Fig. 2 and Fig. S1), with Chr 1 having the highest number of *de novo* genes, followed by Chr6. Notably, the number of *de novo* genes on each chromosome shows no significant relationship with chromosome size or position (Fig. 2), suggesting that the birth of *de novo* genes may be random in *P. persica* “baifeng”.

### Distinctive structures of *de novo* genes (i.e., types I and II genes) and conserved genes

**Figure 3:**
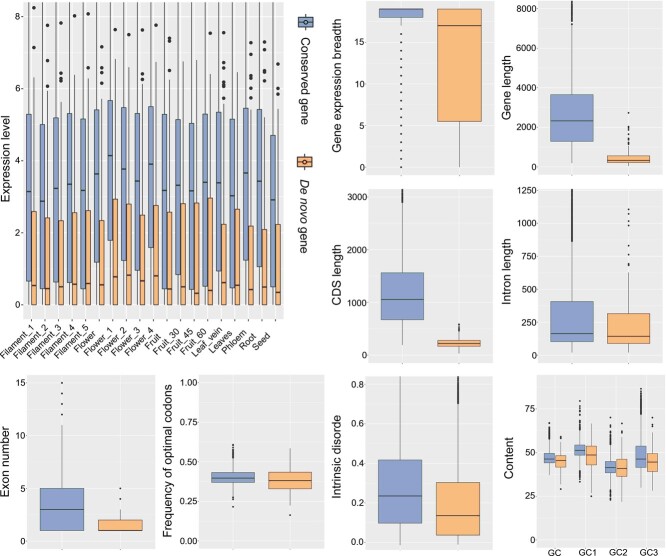
Comparative analysis of gene expression patterns and features between conserved and *de novo* genes. GC represents the overall average GC content spanning all codon positions; GC1 specifies the average GC content at the initial codon position; GC2 details the average GC content at the middle codon position; and GC3 quantifies the average GC content at the terminal codon position.

Given the distinct origins of conserved and *de novo* genes, it is intriguing to explore their unique characteristics. Thus, we first identified 3946 one-to-one orthologs among all tested *Prunus* genomes, which were deemed conserved genes. Subsequently, we analyzed various gene features, including GC content, codon usage patterns, gene lengths, intrinsic disorder (ISD), exon lengths, and gene expression patterns. As shown in Fig. 3, *de novo* genes exhibited significant differences in gene structure with the conserved genes in the genome of *P. persica* “baifeng”. For example, *de novo* genes contained shorter gene lengths, shorter CDS lengths, shorter intron lengths, lower Fop values, lower ISD values, and fewer exon numbers in comparison with the conserved genes in the genome of *P. persica* “baifeng” (Fig. 3). Further analysis of gene structure components revealed that the increased length of conserved genes is primarily due to the integration of additional exons, suggesting that the transition from shorter *de novo* genes to longer conserved genes involves the recruitment of these alternative exons [47]. Similarly, gene duplication significantly contributes to the expansion of conserved gene families, potentially leading to the recruitment of more exons in conserved genes compared to *de novo* genes. In our study, the data showed that *de novo* genes exhibited significantly lower expression levels and narrower expression breadths compared to conserved genes in the genome of *P. persica* “baifeng”, consistent with findings from previous studies [3, 7, 9, 46, 48, 49]. *De novo* genes have lower GC content compared to conserved genes. Detailed analysis of GC content at the first, second, and third codon positions (GC1, GC2, and GC3) revealed higher values in conserved genes. High GC content is often associated with intrinsic structural disorder (ISD) in proteins, suggesting a positive correlation between GC content and ISD values. Our results support this: conserved genes with high GC content show a positive correlation between GC content and ISD values, while *de novo* genes with low GC content exhibit a slight negative correlation with ISD values (Fig. S2).

**Figure 4:**
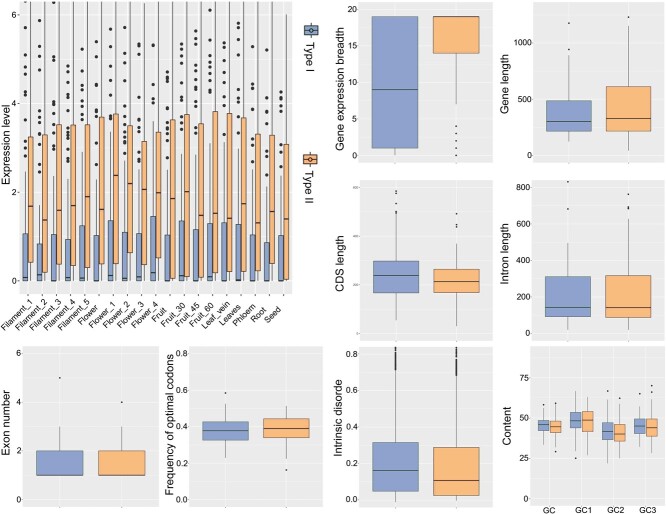
Comparative analysis of gene expression patterns and features between Type I and Type II *de novo* genes. GC represents the overall average GC content spanning all codon positions; GC1 specifies the average GC content at the initial codon position; GC2 details the average GC content at the middle codon position; and GC3 quantifies the average GC content at the terminal codon position.

In our study, *de novo* genes were classified into two types according to their origins. Type I genes exclusively evolved from noncoding sequences initially under neutral evolution, while Type II genes arose from chimeric sequences incorporating minor portions of pre-existing genes subjected to selection pressure [9, 30]. Therefore, we hypothesize that Type I genes, due to their exclusive derivation from noncoding sequences, may more accurately represent the inherent characteristics of *de novo* genes than Type II genes (Fig. 4). To identify distinguishing characteristics between Type I and Type II genes, we further analyzed various gene attributes. As predicted, Type II genes exhibited features more similar to conserved genes in terms of gene expression levels and expression breadth. However, no such pattern was observed for gene length, CDS length, Fop, ISD, and GC content (Fig. 4). Based on these data, we speculate that the selection pressure on noncoding sequences from which *de novo* genes originate does not significantly affect the characteristics of *de novo* genes in the genome of *P. persica* “baifeng”.

### The analysis of gene ontology (GO) suggests type I *de novo* genes originated from sequences with plastid modification functions, while type II were derived from sequences with plant growth and development functions

**Figure 5:**
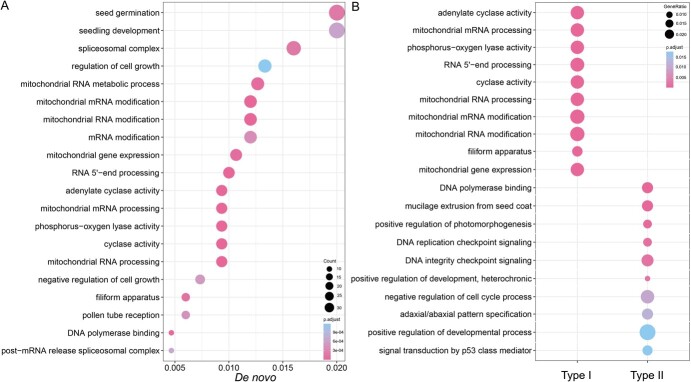
The GO enrichment analysis. (A) Enrichment analysis of GO terms for *de novo* genes. (B) A comparative study of GO term enrichment between Type I and Type II *de novo* genes. This analysis utilized all GO terms from *Prunus de novo* genes, including those associated with sequences located 10-kb upstream and downstream, to evaluate enrichment.

*De novo* genes, which arise from ancestral noncoding sequences, exhibit distinct origins. Type I *de novo* genes incorporate untranslated regions (UTRs) or introns from coding sequences, whereas Type II *de novo* genes integrate exon sequences from ancestral genomes. To determine which ancestral sequences are most likely to evolve into *de novo* genes, we conducted gene ontology (GO) enrichment analysis on the coding sequences (CDSs) that contributed to these *de novo* genes, following the methodology outlined by Song *et al.* [9]. We extracted all genes located within 10 Kb upstream and downstream of the corresponding *de novo* genes and submitted them to eggNOG-mapper to obtain their GO annotation information. GO enrichment analysis using clusterProfiler 4.0 revealed that *de novo* genes derived from noncoding regions are enriched in processes related to plant growth and development, particularly seed germination, photosynthesis, and plastid modification (Fig. 5).

The enriched biological processes were further compared between Type I and Type II genes, revealing that Type I genes, derived from noncoding sequences, are involved in plastid modification. In contrast, Type II genes, originating from chimeric sequences, are enriched in processes related to plant growth and development. This might explain why more Type II genes were identified, as they are enriched in regulatory and single-organism processes (Fig. 5). These findings suggest that different types of *de novo* genes from noncoding sequences have distinct biological functions, with Type I genes associated with plastid modification and Type II genes with growth and development.

### Transcriptional regulatory analysis of *de novo* genes in the genome of *P. Persica* “baifeng”

**Figure 6:**
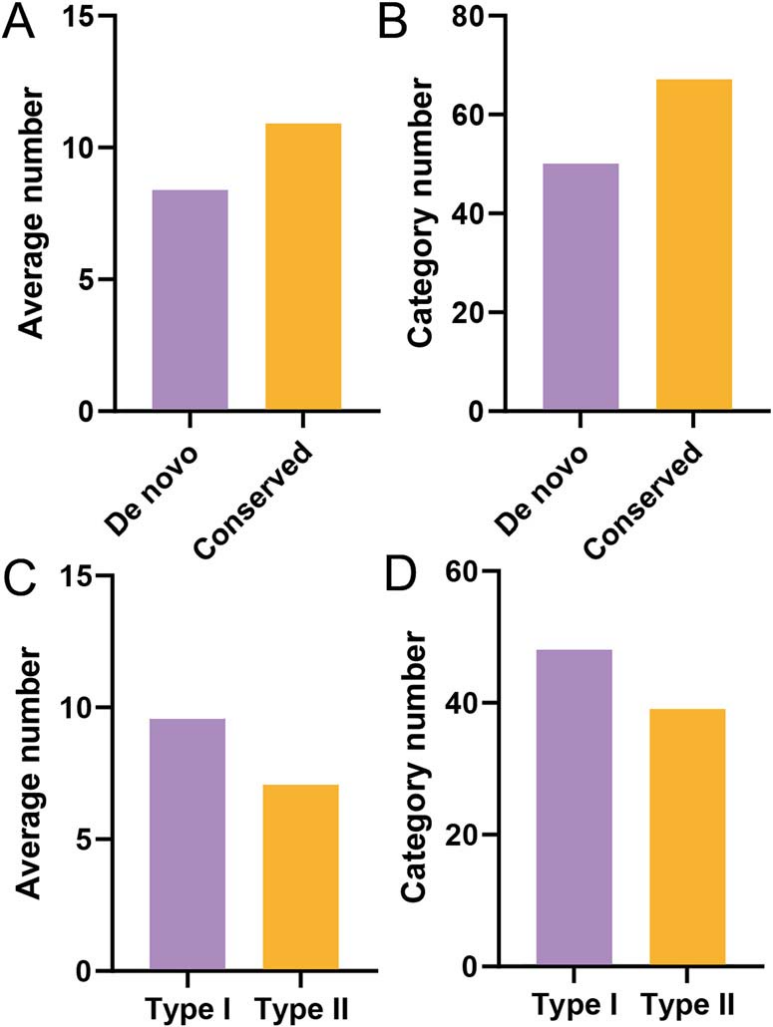
Analysis of transcription factor binding sites. (A) Number of binding sites in *de novo* versus conserved genes. (B) Varieties of binding sites in *de novo* versus conserved genes. (C) Number of binding sites in Type I versus Type II *de novo* genes. (D) Varieties of binding sites in Type I versus Type II *de nov*o genes.

Protein-DNA interactions play a crucial role in regulating various aspects of plant growth, development, and cellular responses to environmental stimuli [50–52]. The divergence of cis-regulatory binding sites (CRBSs) can lead to significant evolutionary changes by altering these protein–DNA interactions [53]. To identify *de novo* genes involved in transcriptional regulation and examine the evolutionary divergence between Type I and Type II genes, we extracted 2-kb upstream sequences from the start codons of all *de novo* genes in the genome of *P. persica* “baifeng”. These sequences were submitted to NSITE [54] and PlantCARE [55] for the prediction of CRBSs. Here, we found that the vast majority (98.3%) of *de novo* genes possess CRBSs, suggesting that these genes have evolved their own transcriptional regulatory sequences in *P. persica* “baifeng”. In summary, 50 categories of CRBSs were detected in the identified *de novo* genes, with each gene containing an average of 8.38 CRBSs. Similarly, CRBSs were found in 99.2% of conserved genes, each exhibiting an average of 10.9 CRBSs (Fig. 6A). Among these conserved genes, all identified CRBSs were categorized into 68 distinct groups (Fig. 6B). Chi-Square test results indicated no significant difference in the categories or numbers of CRBSs between conserved and *de novo* genes. Notably, unique CRBSs were identified in both conserved and *de novo* genes: conserved genes contained elements such as ABF, ALF, AT-hook, BPC, CBF, Cre1, CRR1, GARP, GBP, HD-like, HMG, MYB-like, NF-like, PHR1, PLATZ, SCL, SEBF, SORLIP, SPL, STY, TALE, TEIL, USF, and ZF-like, whereas *de novo* genes included CO-like, KNOX, RAV, SBF, and SRS elements (Tables S3 and S4). Despite the lower number of unique CRBSs compared to common CRBSs, both conserved and *de novo* genes exhibited a similar pattern for common CRBSs, such as MADS, AP2/ERF, WRKY, and MYB elements (Tables S3 and S4). Collectively, these findings confirm that *de novo* genes, which evolved from noncoding regions, have integrated into the existing regulatory networks within the genome of *P. persica* “baifeng”.

Comparative analysis of the CRBSs between Type I and Type II *de novo* genes revealed that these sites were present in the vast majority of Type I (100%) and Type II (96.6%) genes. Each Type I and Type II *de novo* gene contained an average of 9.57 and 7.07 CRBSs, respectively (Fig. 6C and Table S5). The Chi-Square test results indicated no significant difference in the categories (48 for Type I genes vs. 39 for Type II genes) or the number (9.57 for Type I genes vs. 7.07 for Type II genes) of CRBSs between Type I and Type II *de novo* genes (Fig. 6D and Table S5). These findings demonstrate that despite their distinct origins, Type I and Type II *de novo* genes exhibit comparable distributions of TFBSs, suggesting their integration into existing regulatory networks.

### *De novo* gene integration into regulatory networks by co-expression analysis

To obtain the potential functional roles of *de novo* genes, we sequenced the RNA-seq data of different tissues in peach. As shown in Figs S3, 3, and 4, we found that most *de nov*o genes exhibited low-level expression patterns across almost all tissues, which is consistent with previous descriptions of *de novo* gene expression patterns [4, 9, 49]. More strikingly, *de novo* genes were predominantly detected in reproductive tissues or developmental stages, despite their lower expression levels compared to conserved genes. This finding is consistent with observations that over 50% of *de novo* genes in *Caenorhabditis elegans* are expressed during dauer stages. Similarly, Begun *et al.* [56] demonstrated that *de novo* genes in *Drosophila* are implicated in male reproductive functions. Collectively, these results reinforce the hypothesis that *de novo* genes play crucial roles in reproductive processes.

*De novo* genes integrated into gene interaction networks can reveal their novel functional roles [33, 57]. Using weighted gene coexpression network analysis (WGCNA) [58], we analyzed correlations between genes across different transcriptome samples and clustered genes with similar expression patterns into corresponding modules. We grouped 23 240 genes into 25 distinct modules, with sizes varying from 69 to 3131 genes (Fig. S4). Except for the darkred, darkturquoise, and royalblue modules, all other modules contain both old and *de novo* genes. Among these, *de novo* genes are primarily found in six modules (black; Fig. S5, yellow; Fig. S6, blue; Fig. S7, pink; Fig. S8, purple; Fig. S9, and turquoise; Fig. S10) with each of these modules containing more than 10 *de novo* genes (Table S6). These specific networks encompass between 1005 and 3131 genes and are typically enriched with genes involved in various plastid modification functions, synthetic and catabolic pathways, as well as stress response (Figs S5–S10). Our results indicate that *de novo* genes are co-expressed with a large number of old genes in different tissues or developmental stages of peach, and are subsequently integrated into regulatory networks, where they play significant roles in growth, development, and stress responses. These conclusions are further supported by the QTL results (Tables S7 and S8).

### *De novo* genes occur in mendelian trait loci (MTL) and quantitative trait loci (QTL) with environmental selection stresses and growth and development

To further explore the potential functions of *de novo* genes, we used QTLs and MTLs data to provide evidence for the roles of these genes in the growth and development of *P. persica* “baifeng”. Although not all genes mapped to QTLs or MTLs were key or causal genes, these genes located in QTL or MTL regions had potential functions related to the corresponding QTLs or MTLs. In peach, many previously published manuscripts have identified QTLs or MTLs associated with related-traits such as seed size, seed coat color, growth habit, and environmental selection stresses [59–86]. In our study, we identified *de novo* genes from QTLs or MTLs in *P. persica* “baifeng” based on their chromosomal locations in the corresponding *Prunus* genomes. Finally, 25 *de novo* genes, including 16 Type I and 9 Type II genes, were mapped onto QTLs or MTLs involved in plant growth, development, or fruit quality control, suggesting that these *de novo* genes potentially regulate physiological changes (Table S7). Remarkably, two of the identified *de novo* genes were situated within at least two MTLs or QTLs, suggesting potential pleiotropic functions (Table S8).

## Discussion

*De novo* genes are a driving force for phenotypic evolution [1, 33, 56]. Understanding their origins is a fundamental biological question [9, 20, 27, 87, 88]. Initial pioneering work identified several *de novo* genes in *Drosophila* [20, 21]. Subsequently, *de novo* genes have been discovered in yeast, humans, primates, *Plasmodium*, rice, Arabidopsis, and peanuts [5, 8, 9, 12, 16, 22, 32, 89]. The discovery of candidate *de novo* genes is often achievable with concerted effort, making this a burgeoning area of research. However, identifying *de novo* genes poses several challenges, including species-dependent variability in the number of identified genes [18, 30]. Ideally, comparing genome sequences across a broad sample of taxa enhances the accuracy of *de novo* gene identification in specific plant genomes [9]. Peach (*P. persica*) is an economically important perennial fruit crop worldwide. The sequencing of whole genomes for many *Prunus* species [41–45] has facilitated the identification of *de novo* genes in *P. persica* “baifeng”. In this study, we identified at least 178 *de novo* genes evolved from noncoding sequences, with 158 of these genes expressed in at least one tissue.

Researchers have proposed several models for *de novo* gene birth, including the adaptation following the neutral sequence evolution model, the grow slow and moult model, and the preadaptation model [7, 16, 90, 91]. Within these models, the proto-gene concept posits that the emergence of *de novo* genes is a gradual process, facilitated by “proto-genes” that evolve from noncoding sequences [7, 27]. This model posits that some proto-genes are occasionally retained by natural selection and gradually evolve to acquire functional roles [27]. Initially, these noncoding sequences exhibit translation and transcription in a selectively neutral manner [7, 27, 92]. Over time, they mature under selection pressure, providing adaptive advantages and eventually becoming *de novo* genes [7, 13, 27]. Consequently, proto-genes exhibit characteristics intermediate between nongenes and fully functional genes, influencing their sequence features and expression patterns [7, 13, 27]. These data suggest that conserved and *de novo* genes can be distinguished by analyzing characteristics such as evolutionary patterns, gene expression profiles, and gene structures. In our study, we compared the characteristics between conserved genes and *de novo* genes, finding that *de novo* genes contain shorter sequences, narrower gene expression breadths, and lower expression levels compared to conserved genes, consistent with the findings from previous studies [7, 13, 27, 46, 57, 92]. Current research on *de novo* genes mainly focuses on their birth, while the classification, expression patterns, evolutionary, and structural characteristics of different classes of *de novo* genes are rarely explored. Therefore, our study mainly identified existing *de novo* genes without revealing the birth of *de novo* genes. Here, our findings indicate that Type II genes exhibit similarities to conserved genes in terms of expression levels and breadth, a conclusion supported by Song *et al.* [9]. However, for other structural features, such as ISD values and GC content, these two different types of genes exhibited complex phenomena, suggesting that whether the noncoding sequences from which *de novo* genes originate undergo selection have no effect on the features of *de novo* genes in the genome of *P. persica* “baifeng”.

Type I genes arise entirely from noncoding sequences. Type II genes originate from a small number of pre-existing genes [9]. The promoters of Type I *de novo* genes which evolved entirely from noncoding regions instead of duplicated from a small number of pre-existing gene promoters might be expected to have a different frequency of CRBSs than in Type II genes. Type II genes might inherit CRBSs from their pre-existing genes, thereby benefiting from established regulatory mechanisms [93]. Our findings reveal that the promoters of *de novo* genes exhibit comparable numbers and categories of CRBSs to those of conserved genes. Furthermore, the promoters of Type I and Type II *de novo* genes also display similar numbers and categories of CRBSs. CRBSs can quickly establish themselves after a new gene emerges, likely due to the frequent turnover of CRBSs, a well-documented phenomenon in eukaryotic cis-regulation [94, 95]. For instance, in yeast, the frequent acquisition of CRBS in new genes is crucial for regulatory evolution [94, 95]. Papp *et al.* [94] observed that the number of CRBSs in new genes created by duplication remained stable over time, while shared motifs from conserved ancestral genes decreased. This pattern suggests a balance between acquiring new CRBSs and losing those from a small number of pre-existing genes [94, 96].

A crucial question in the study of gene evolution is determining which types of noncoding sequences have the potential to give rise to *de novo* genes. In plants, the plastid genome plays a significant role in the emergence of *de novo* genes within the nuclear genome [97–99]. For instance, the mitochondrial genome is a frequent source of *de novo* genes. Due to the high rate of rearrangement in plant mitochondrial genomes, the occurrence of *de novo* genes from the mitochondrial genome is often 2–10 times higher than from the nuclear genome [99–101]. In many plant lineages, intergenomic gene transfer events facilitate the integration of mitochondrial genome segments into the nuclear genome [98]. Our study revealed that Type I *de novo* genes arise entirely from noncoding sequences associated with plastid modification functions, while Type II genes are derived from sequences related to reproductive functions. Supporting this, previous studies on the *A. thaliana* genome have shown similar patterns, where *de novo* genes originating from mitochondrial DNA preferentially code for mitochondrial-targeting peptides compared to those from intergenic DNA [98, 99].

*De novo* genes drive species-specific evolution [102–104], though their functions often remain unclear. Evidence indicates that *de novo* genes are more condition-specific or tissue-specific in their expression compared to conserved genes [9, 104, 105]. To explore their potential functions, we analyzed RNA-seq expression data. Sequencing the transcriptomes of different tissues and developmental stages in peach revealed that *de novo* genes exhibit higher expression levels in reproductive tissues compared to other tissues. Indeed, the researchers observed relatively high expression of *de novo* genes in reproduction tissues or during development in humans, *Drosophila*, mice, and bamboo and found that these highly expressed genes play important roles in these tissues [27, 33, 49, 57]. *De novo* genes have been identified that may have fused into plant-wide regulatory networks [9, 33, 57]. For example, Jin *et al.* [33] found that *de novo* genes from woody bamboo were involved in the shoot rapid growth by WGCNA analysis. *De novo* genes from rice play important roles in resistance to *Xanthomonas oryzae* pv. *Oryzae* infection [36]. In *Mus musculus* and *D. melanogaster*, *de novo* genes are involved in testis development [25, 106]. In our study, we found that *de novo* genes are involved in plastid modification processes and growth and development. However, there is limited evidence that *de novo* genes integrate into existing regulatory networks, leading to phenotypic changes and novel traits in *P. persica* “baifeng”. Using WGCNA and QTL analysis, we demonstrated interactions between *de novo* genes and conserved genes, finding enrichment in genes related to plant growth, development, and fruit quality control. These results suggest that identified *de novo* genes play important roles in peach growth and development by integrating into existing regulatory networks.

## Conclusions

In our study, we identified and classified *de novo* genes in the genome of *P. persica* “baifeng” and revealed that *de novo* genes differ from conserved genes in their evolutionary characteristics, gene features, and expression patterns, which was supported by the published papers. The GO analysis revealed that Type I *de novo* genes, originating from noncoding sequences, are linked to plastid modifications, whereas Type II genes arise from sequences associated with plant growth and development. Transcriptome sequencing revealed that these *de novo* genes have been integrated into existing regulatory networks, leading to phenotypic changes and novel traits in *P. persica* “baifeng”. This peach variety exhibits many unique phenotypes compared to other *Prunus* species, potentially due to *de novo* gene birth, although further research is needed. Our study is the first to identify *de novo* genes in peach, providing a foundation for future research on their evolution and function in *Prunus*.

## Materials and methods

### Transcriptome sequencing and analysis

The different tissues of *P. persica* “baifeng” were obtained from Wuhan Botanical Garden of the Chinese Academy of Sciences, Wuhan, China. Total RNA was extracted using the RNAprep Pure Plant kit (Tiangen, Beijing, China), and genomic DNA contamination was removed by treatment with RNase-free DNaseI (Takara). The NEB-Next® Ultra™ RNA Library Prep Kit for Illumina® (New England Biolabs, Beverly, MA, USA) was used to construct the cDNA library, as described by Cao *et al.* [51]. Qubit 3.0 Fluorometer (Life Technologies) was used to carry out the RNA-Seq libraries quality control (QC) assays. Illumina HiSeq Xten platform was used to sequence these constructed RNA-Seq libraries. Raw data were trimmed and then mapped to the reference genome, as described by Jiang *et al.* [107]. Uniquely mapped reads from each library were processed with StringTie (v2.2.0) with default settings for transcript assembly [108], using the annotated transcripts of *P. persica* “baifeng” as reference sequences. The transcripts from various tissues were merged using StringTie Merge. Transcript quantification was recalculated as fragments per kilobase of transcript per million mapped reads (FPKM) using the merged transcript structures in StringTie. GffCompare was used to identify novel transcripts and their predicted isoforms [109]. As no alternative splicing events were identified in the *de novo* genes, only the representative transcript with the longest coding sequence (CDS) in each gene was used for all subsequent analyses. In our study, we defined transcripts as not expressed when their FPKM values were zero across different biological replicates, as described by Song *et al.* [9].

### *De novo* genes identification in *P. Persica* “baifeng”

The genomes of *P. persica* “CN14”, *P. persica* “Batsch”, *P. dulcis*, *P. persica* “Chinese Cling”, *P. ferganensis*, *P. mira*, *P. dulcis*, and *P. davidiana* were downloaded from GDR (https://www.rosaceae.org/) [41–45, 110, 111]. We used the protein sequences of *P. persica* “baifeng” as queries against protein sequences from *P. persica* “CN14”, *P. persica* “Batsch”, *P. dulcis*, *P. persica* “Chinese Cling”, *P. ferganensis*, *P. mira*, *P. dulcis*, and *P. davidian* using a local BLASTp program (with E-value ≤10^−5^) [112]. We only retained these genes contained in *P. persica* “baifeng” genome to be considered as candidate *de novo* genes. These candidate DNA sequences were used to map onto genome sequences of *P. mira* and *P. dulcis* with BLAT [113]. Finally, we determined these genes as putative *de novo* genes if candidate DNA sequences aligned onto noncoding sequences, as described by Song *et al.* [9]. To ensure accurate results, we aligned the sequences from all *Prunus* species with the putative *de novo* genes, avoiding the influence of noncoding regions that may contain unannotated ORFs.

### Characterization of *de novo* genes

The genes of *P. persica* “baifeng” were considered conserved genes if they have one-to-one homologous genes in other all related species: *P. persica* “CN14”, *P. persica* “Batsch”, *P. dulcis*, *P. persica* “Chinese Cling”, *P. ferganensis*, *P. mira*, *P. dulcis*, and *P. davidian*, using BLASTp with E-value threshold of ≤10^−5^. Then we analyzed the characteristics, including GC content, intrinsic disorder (ISD), frequency of optimal codons (Fop), exon number and length, gene length, gene expression breadth, and gene expression level, between conserved and *de novo* genes in *P. persica* “baifeng”. Expression levels and breadths for genes were analyzed across 19 tissues. We applied a log2 transformation to the FPKM+1 values for normalizing the expression of conserved and *de novo* genes. The breadth of gene expression was quantified by the count of tissues expressing each gene. We retrieved data on gene, exon, intron, and CDS lengths, along with exon counts, from a GFF3 file via TBtools (version 2.096) [114]. Codon usage bias, expressed as the Fop, was determined using CodonW (version 1.4.2), with values ranging from 0 to 1 (http://codonw.sourceforge.net) [115]. GC content at the first, second, and third codon positions (GC1, GC2, GC3) was computed using a proprietary Perl script, following protocols by Song *et al.* [9]. Protein stability, measured by the intrinsic disorder score (ISD) from 0 (stable) to 1 (unstable), was assessed using the iupred3.py script from the IUPred website (https://iupred.elte.hu/) [116].

### Cis-regulatory binding sites (CRBSs) of *de novo* genes

Transcriptional regulation involves one or more transcription factors binding to the promoter region of a gene and is controlled by the coordinated binding of these factors [50]. Sequences within the 2-kb region upstream of the start codon (ATG) of a gene can predict its transcription factor binding site or cis-regulatory binding sites (CRBSs). To gain the CRBSs of conserved and *de novo* genes, we used the TBtools (version 2.096) to extract the 2-kb upstream sequences [114]. Subsequently, the PlantCARE (https://bioinformatics.psb.ugent.be/webtools/plantcare/html/) [55] and NSITE (www.softberry.com/) [54] were used to predict the CRBSs with a *p*-value threshold of 0.05. The predicted CRBSs were considered statistically significant when they met the following two criteria: (1) the total number of predicted cis-regulatory elements exceeds the upper limit of the 95% confidence interval and (2) the expected number of predicted cis-regulatory elements was within a set threshold [9, 54]. Finally, we used the PlantTFDB (https://planttfdb.gao-lab.org/) to annotate the predicted transcription factors [117].

### Functions analysis of *de novo* genes

The function and evolution of *de novo* genes have been the focus of researchers [9, 11, 30]. To obtain what kind of sequence regions may form *de novo* genes, the 10-kb sequences downstream and upstream of *de novo* genes that corresponded noncoding sequences in genomes were extracted, respectively. Based on the gff3 files, we obtained the CDS sequences of all genes in the extracted sequence. The eggNOG-mapper (http://eggnog-mapper.embl.de/) was used to obtain GO terms for all extracted genes [118]. The clusterProfiler (version 4.0.5) was used to carry out the GO enrichment for these genes within 10-kb sequences downstream and upstream [119]. Finally, we used Fisher’s exact test to determine statistically significance enriched GO terms with a *p*-value threshold of 0.05.

### Gene coexpression analysis

We used the WGCNA to carry out the weighted correlation network analysis for different tissues in R (version 4.2.1) [120]. The corresponding gene information for all modules was extracted for further analysis, respectively. In our study, we selected the module with the highest MS score among all modules and defined it as the key module for further analysis, as described by Jin *et al.* [33].

### MTL and QTL data

The QTL and MTL data were also used to infer the functions of identified *de novo* genes in the genome of *P. persica* “baifeng”. To date, more and more QTLs and MTLs have been identified with associating with controlling growth and development, seed coat color, seed size, and growth habit-related traits and responses to biotic stress in peach [59–86]. We determined the chromosomal locations of QTLs and MTLs using the corresponding *Prunus* genomes. The QTLs and MTLs were extracted from corresponding *Prunus* genomes. Then, the extracted QTLs were mapped onto the genome of *P. persica* “baifeng” using a local BLAT search [113]. Finally, we identified *de novo* genes in the aligned regions using chromosomal locations, as described by Song *et al.* [9].

## Supplementary Material

Web_Material_uhae252

## Data Availability

The RNA-seq project of peach (*P. persica*) has been deposited at the BIG Data Center (https://bigd.big.ac.cn/gsa) under the accession: PRJCA011988.
