# Supplementary figures and images for: De novo gene integration into regulatory networks via interaction with conserved genes in peach
Source: Hortic Res. 2024 Sep 5;11(12):uhae252. doi: 10.1093/hr/uhae252 (PMC11630308; doi:10.1093/hr/uhae252)

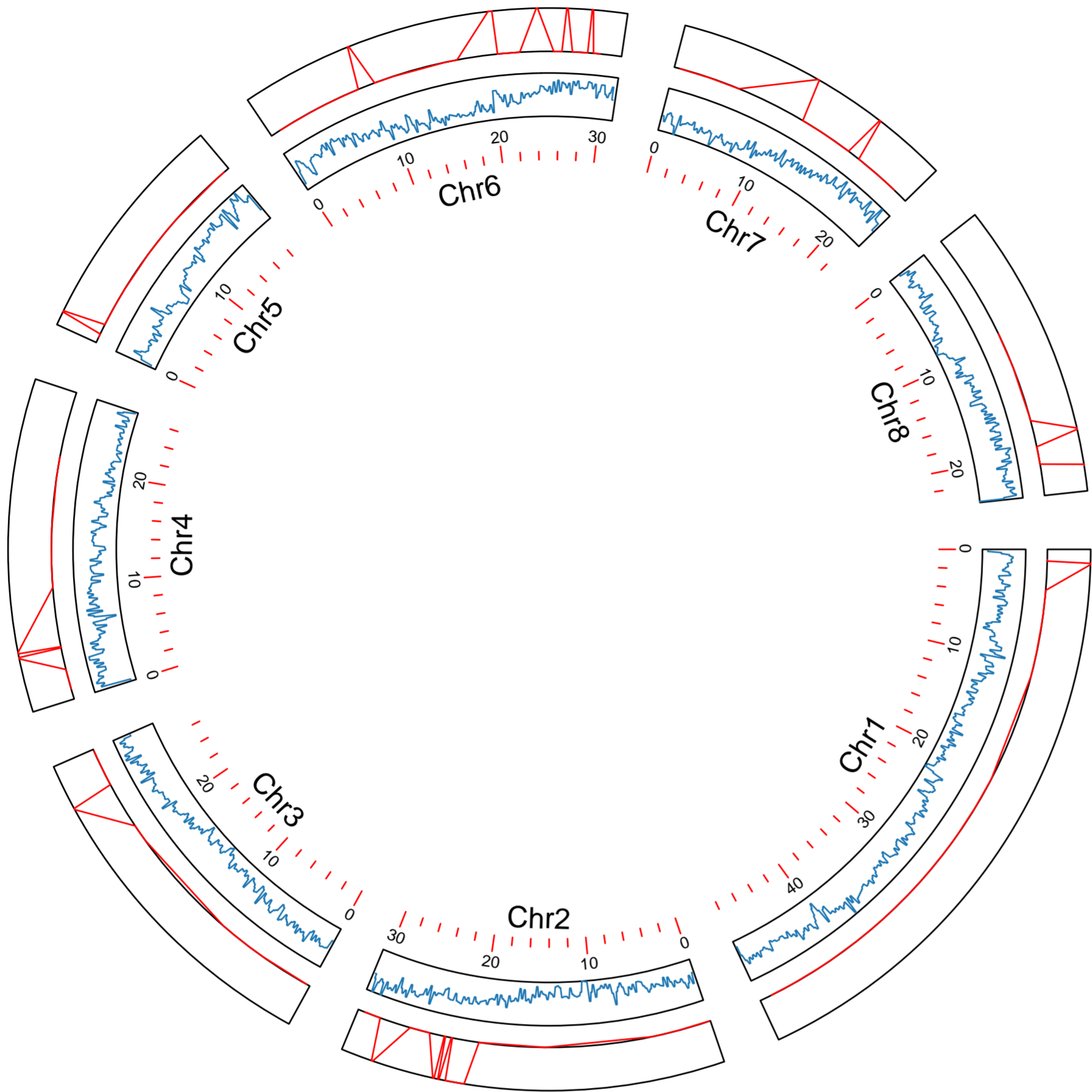

Supplement: Web_Material_uhae252 [file web_material_uhae252.zip › Figure S1.pdf]

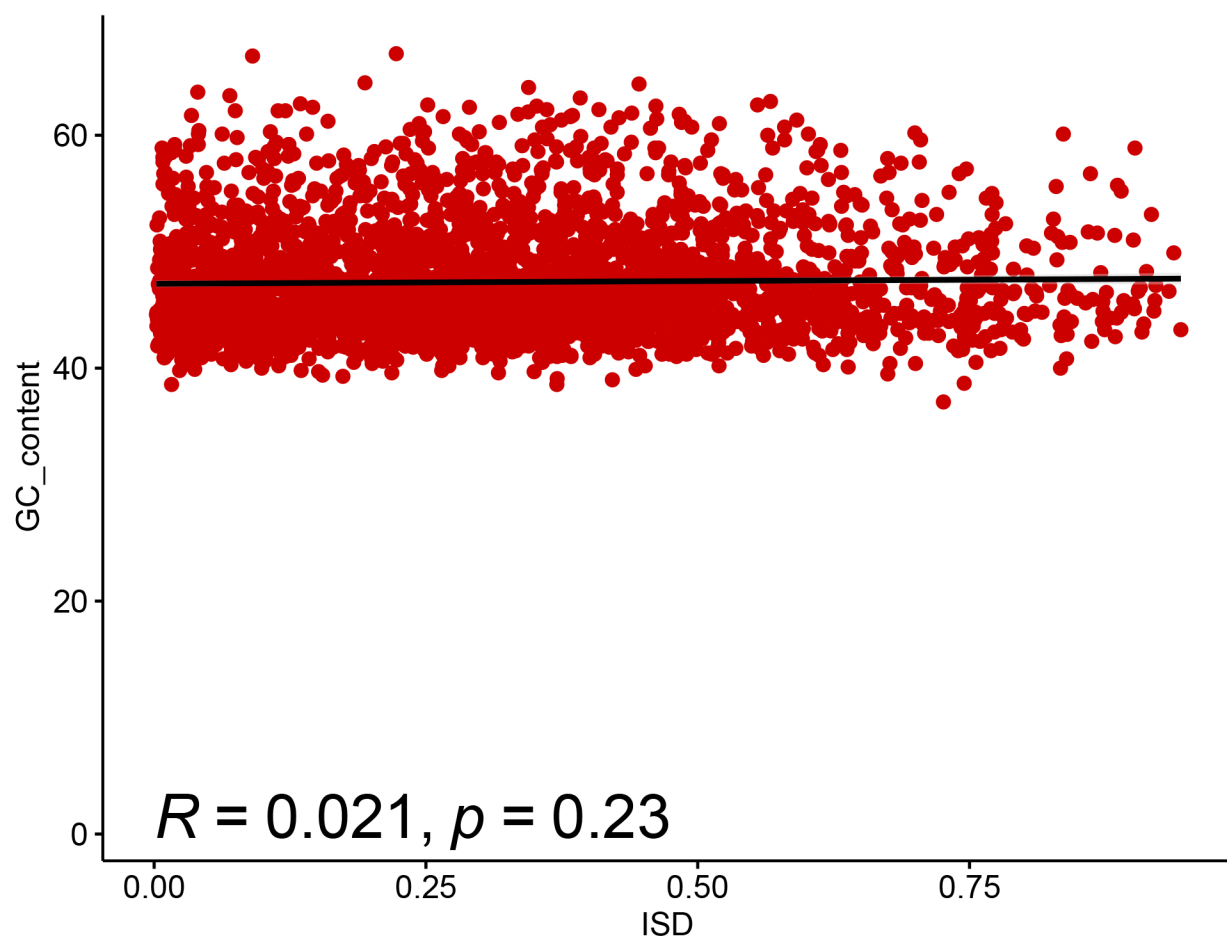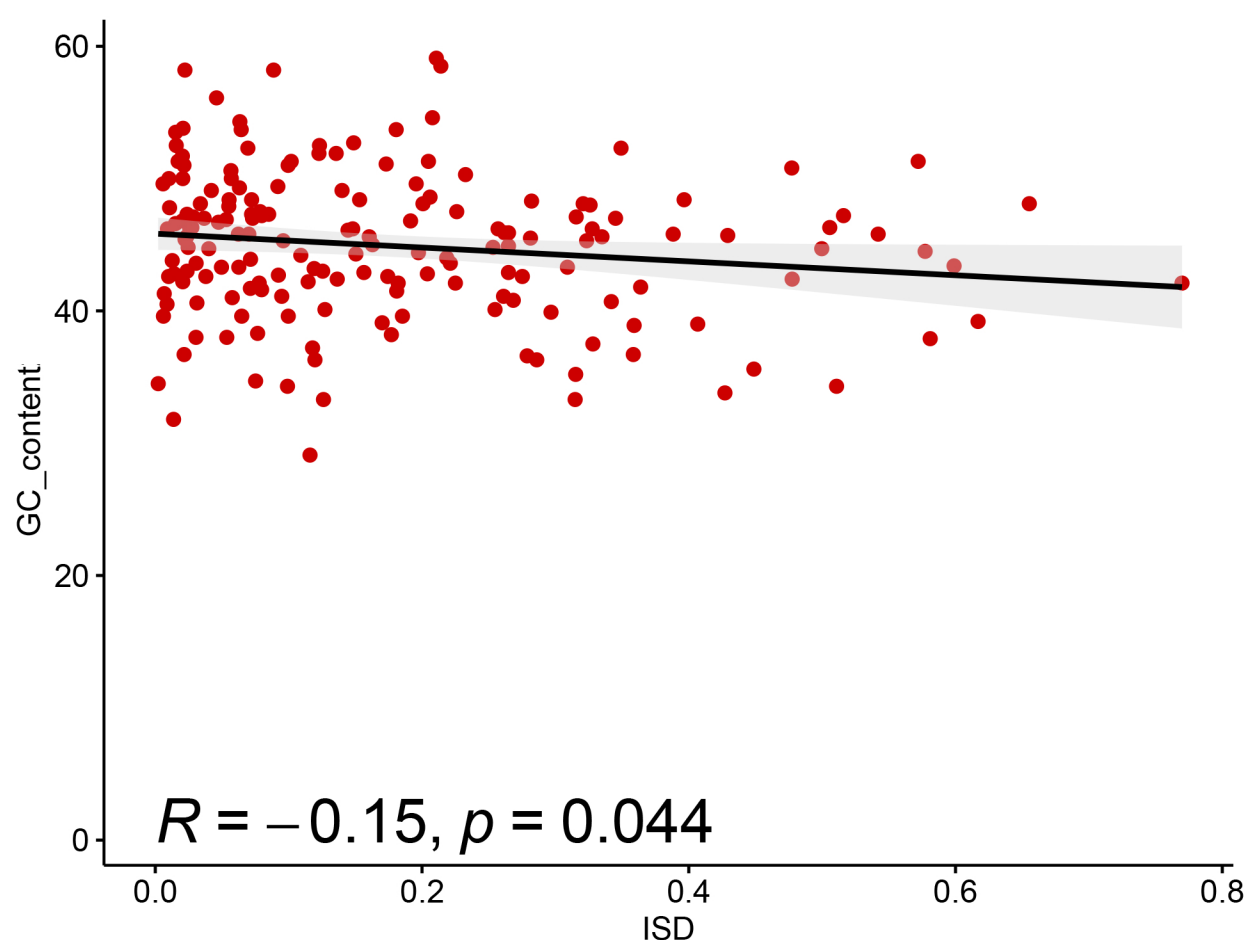

Supplement: Web_Material_uhae252 [file web_material_uhae252.zip › Figure S2.pdf]

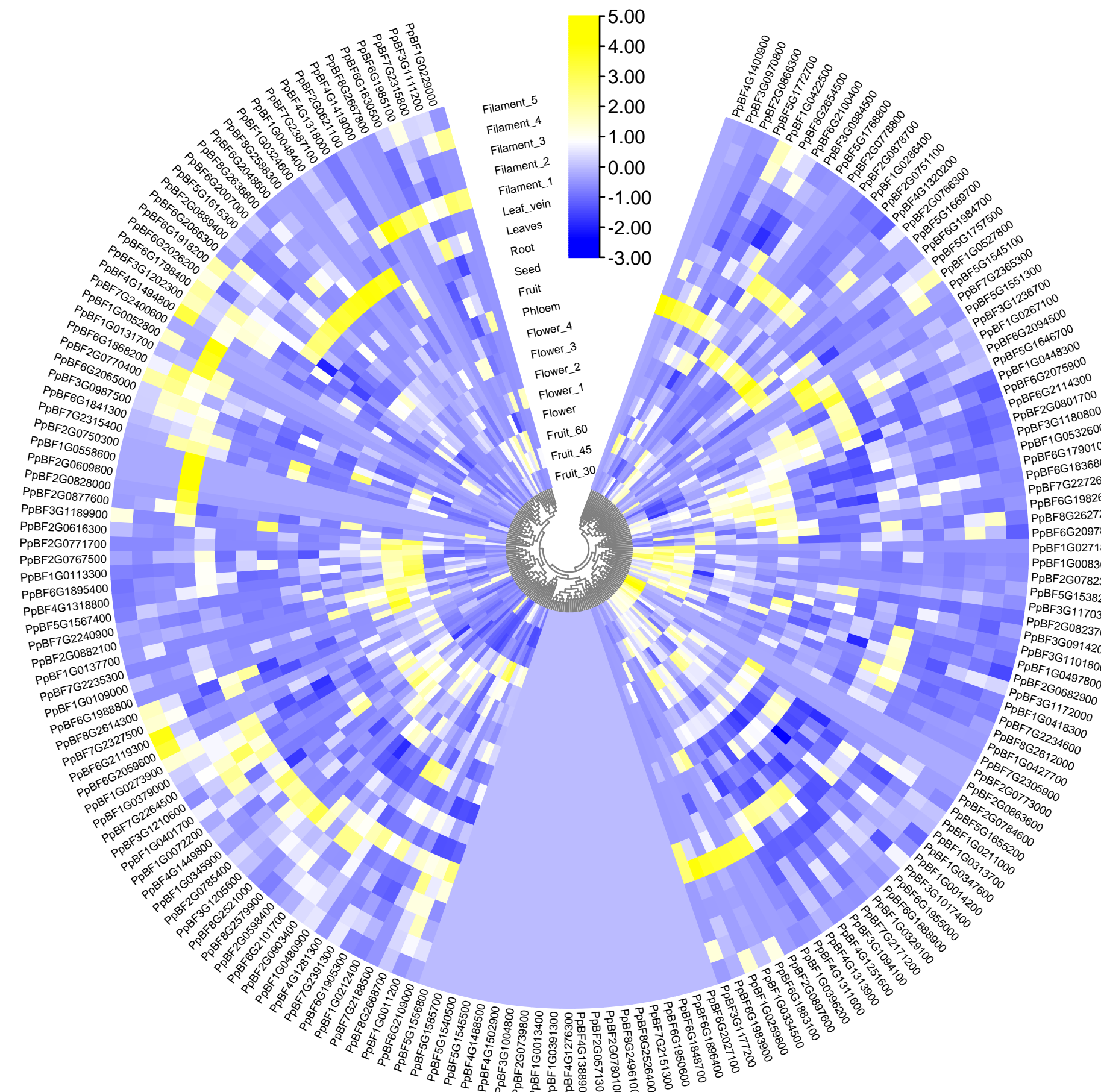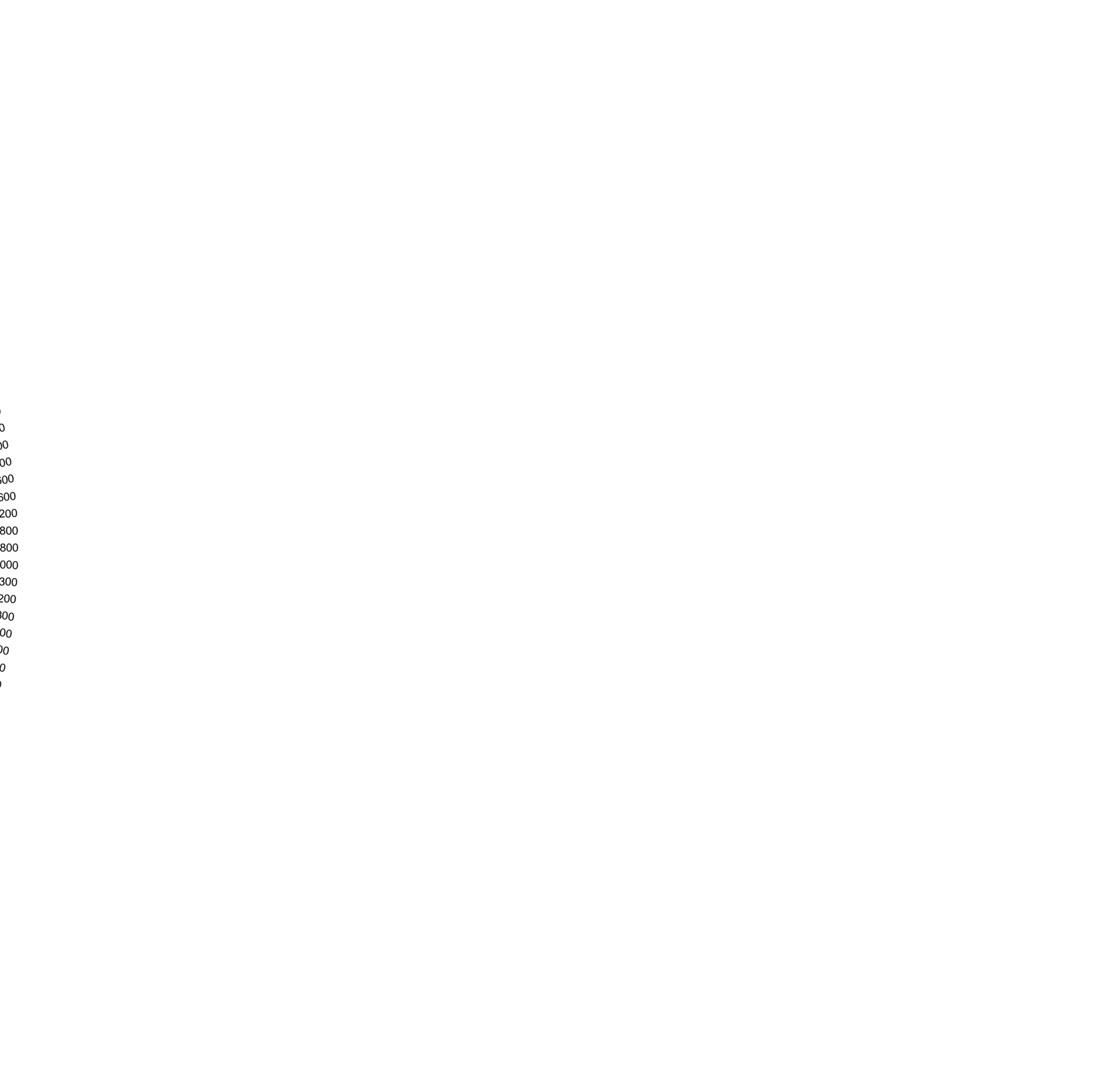

Supplement: Web_Material_uhae252 [file web_material_uhae252.zip › Figure S3.pdf]

### Cluster Dendrogram

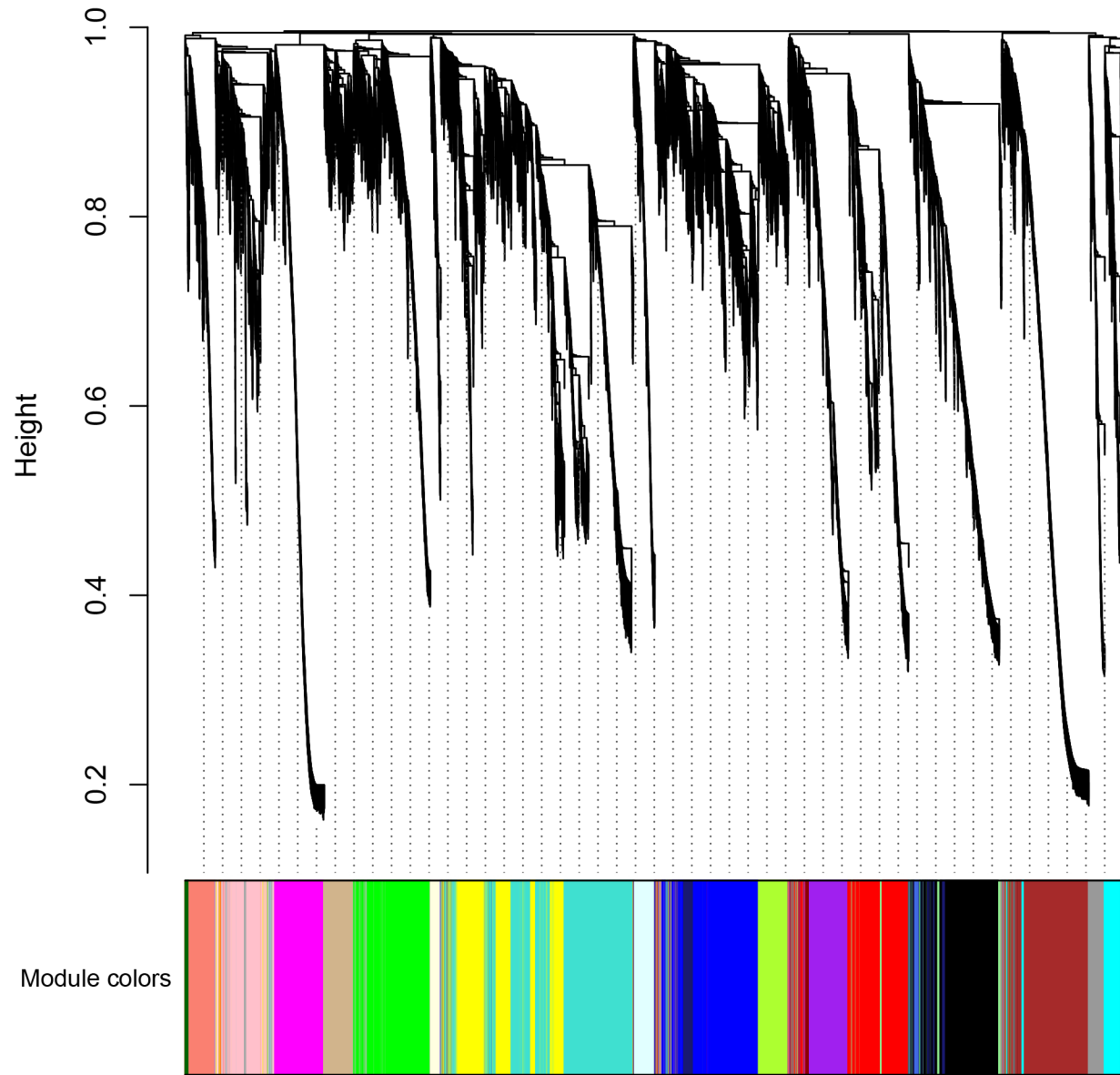

### Eigengene adjacency heatmap

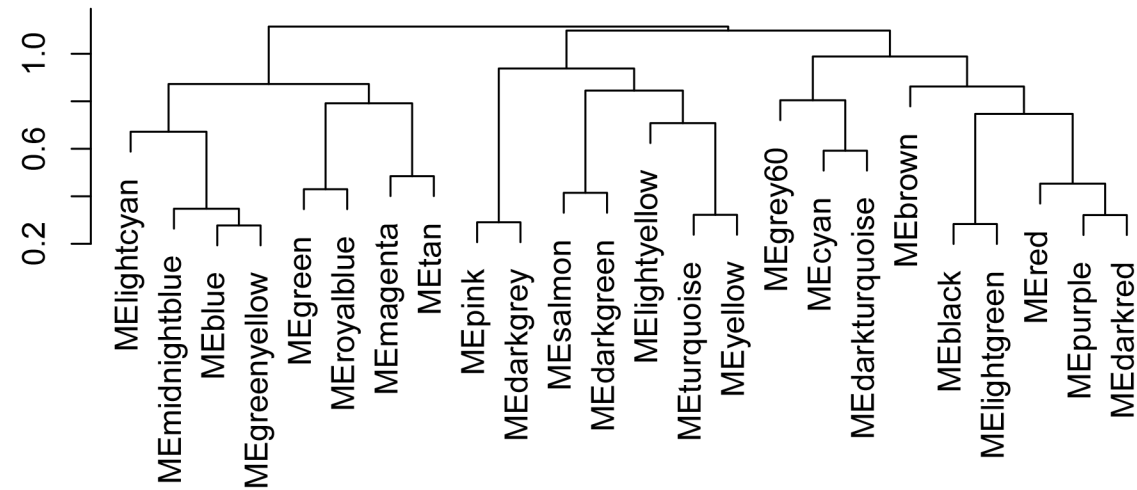

### Eigengene adjacency heatmap

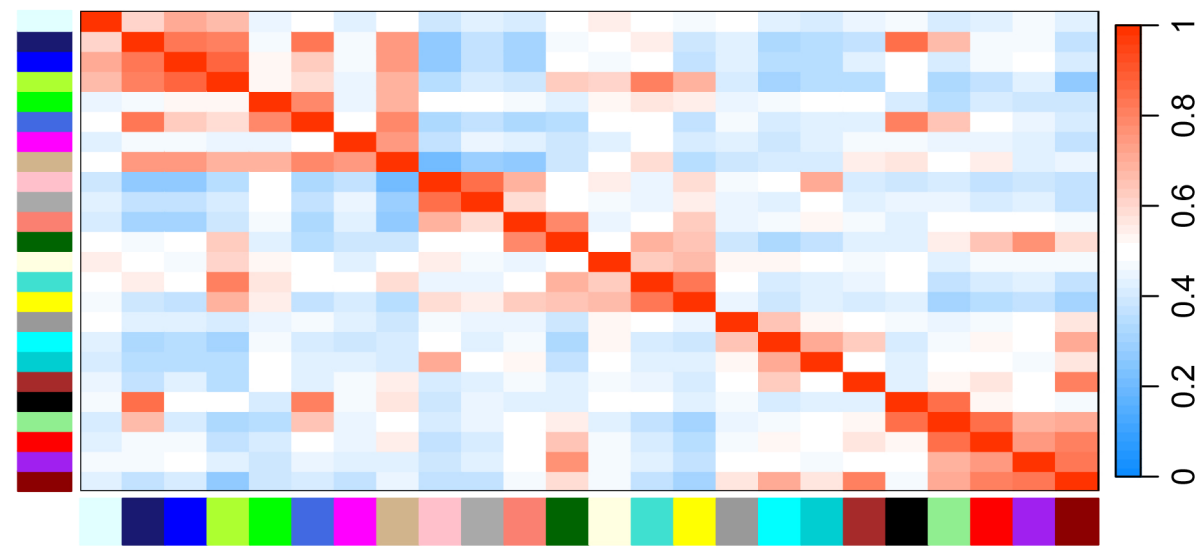

Supplement: Web_Material_uhae252 [file web_material_uhae252.zip › Figure S4.pdf]

black Top 20 GO enrichment result

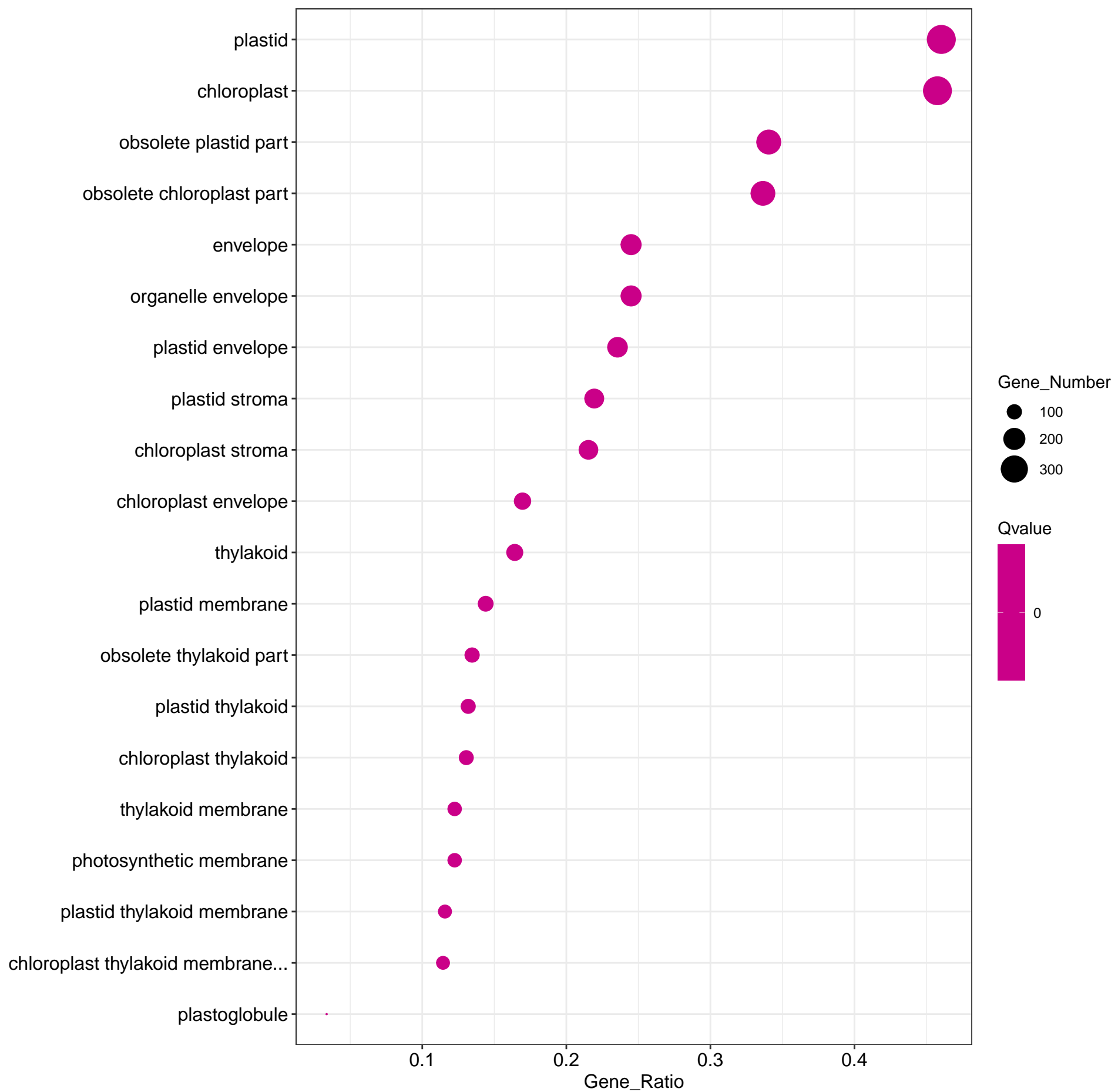

Supplement: Web_Material_uhae252 [file web_material_uhae252.zip › Figure S5.pdf]

yellow Top 20 GO enrichment result

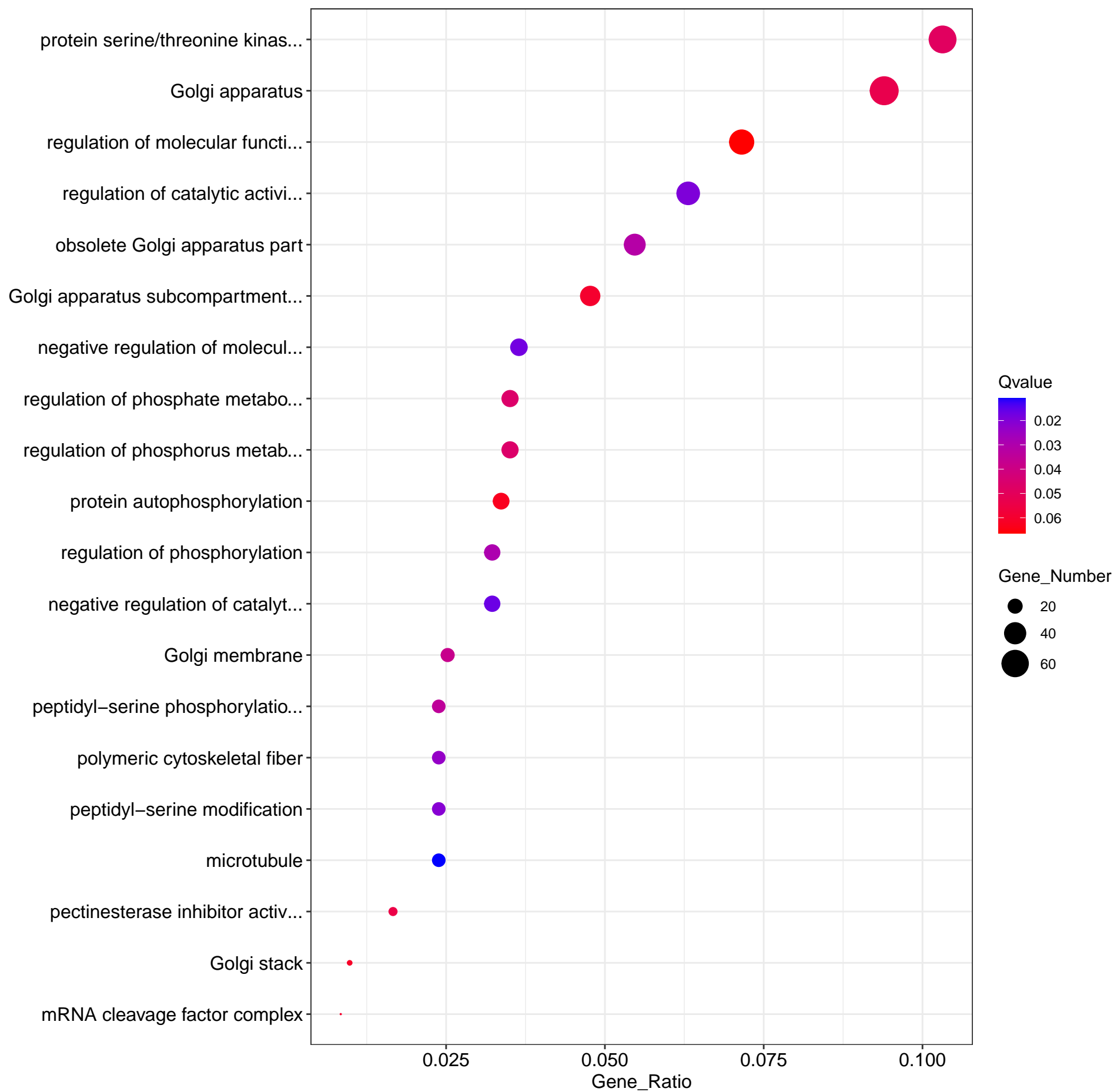

Supplement: Web_Material_uhae252 [file web_material_uhae252.zip › Figure S6.pdf]

blue Top 20 GO enrichment result

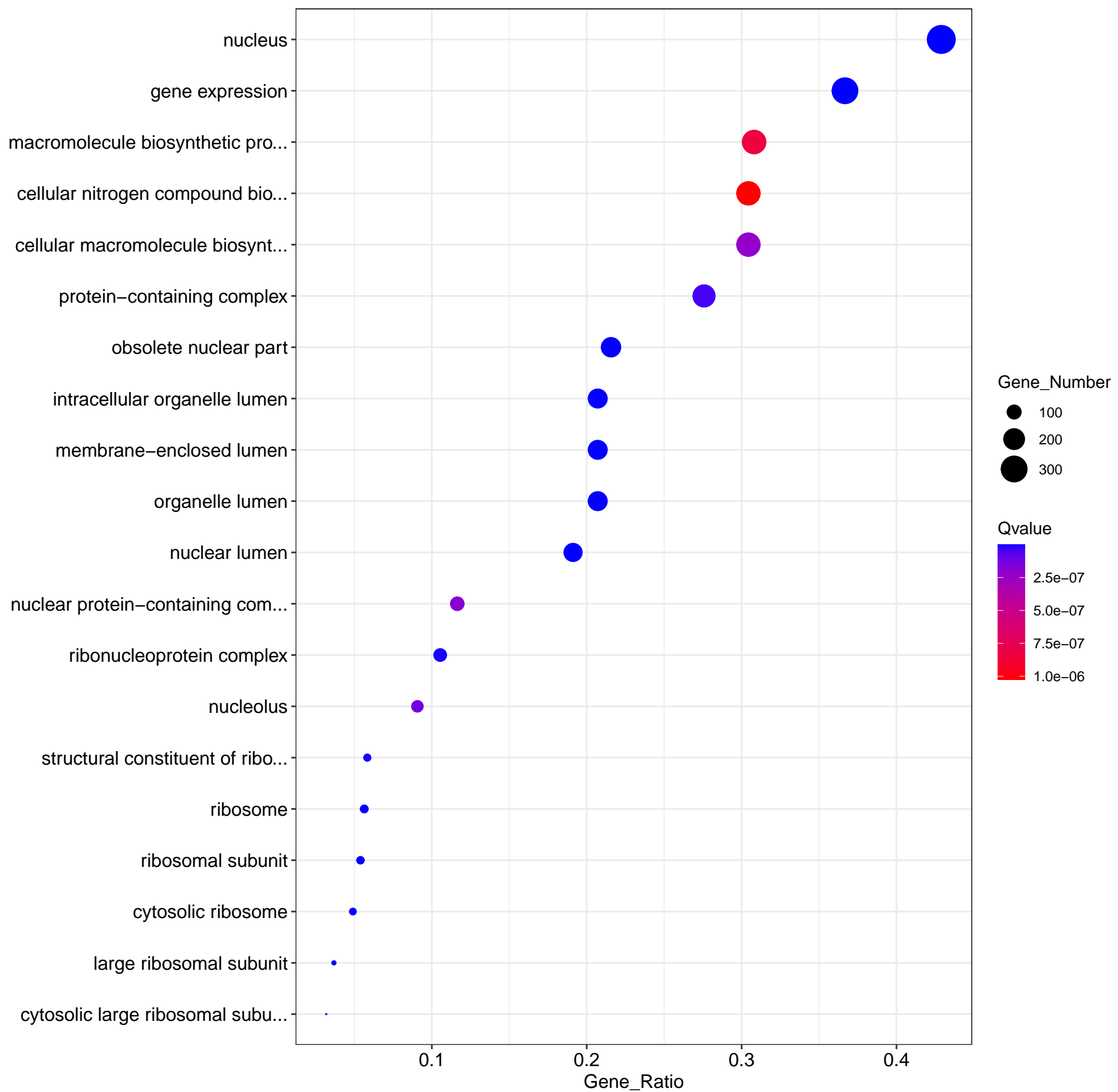

Supplement: Web_Material_uhae252 [file web_material_uhae252.zip › Figure S7.pdf]

pink Top 20 GO enrichment result

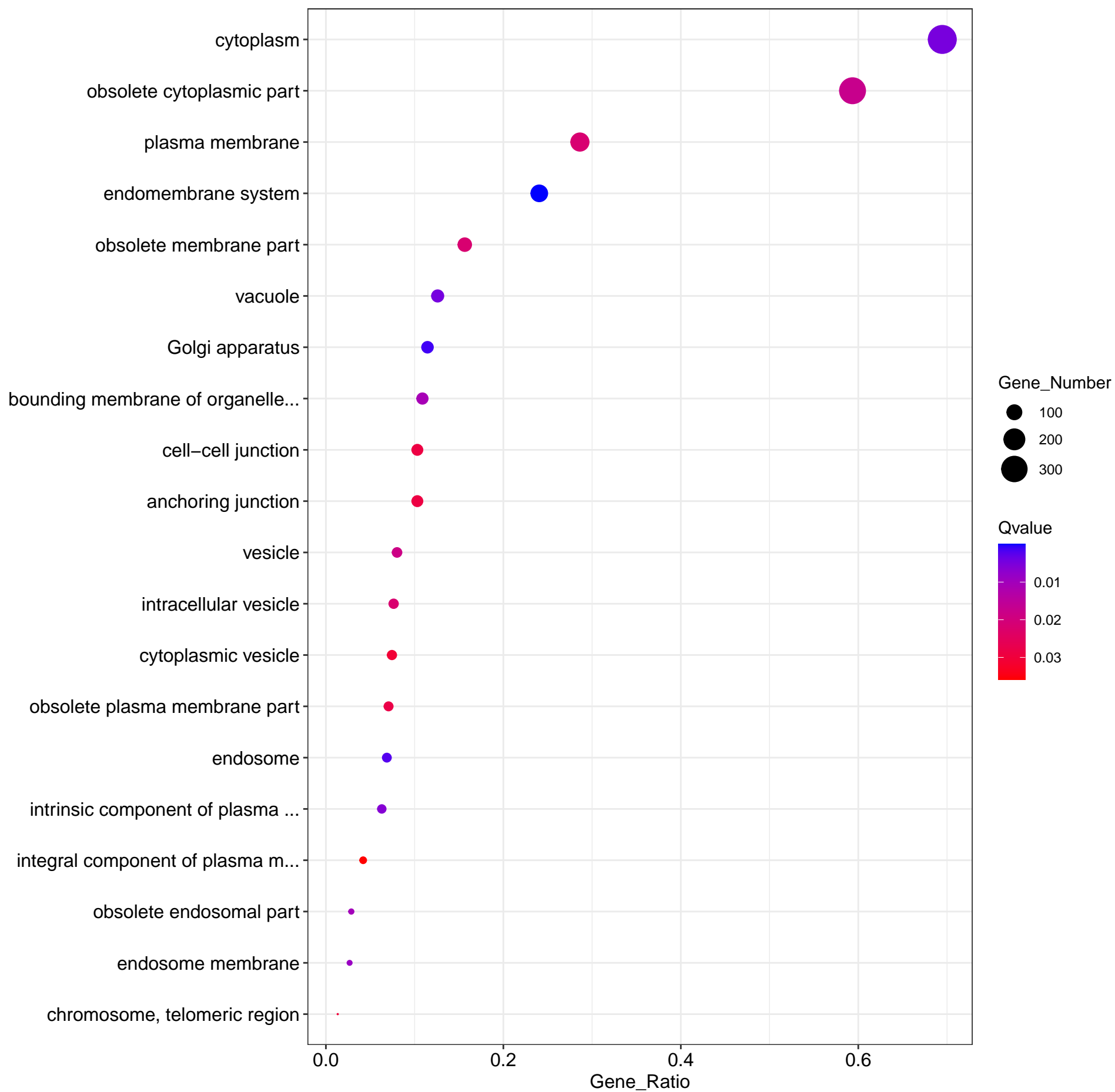

Supplement: Web_Material_uhae252 [file web_material_uhae252.zip › Figure S8.pdf]

purple Top 20 GO enrichment result

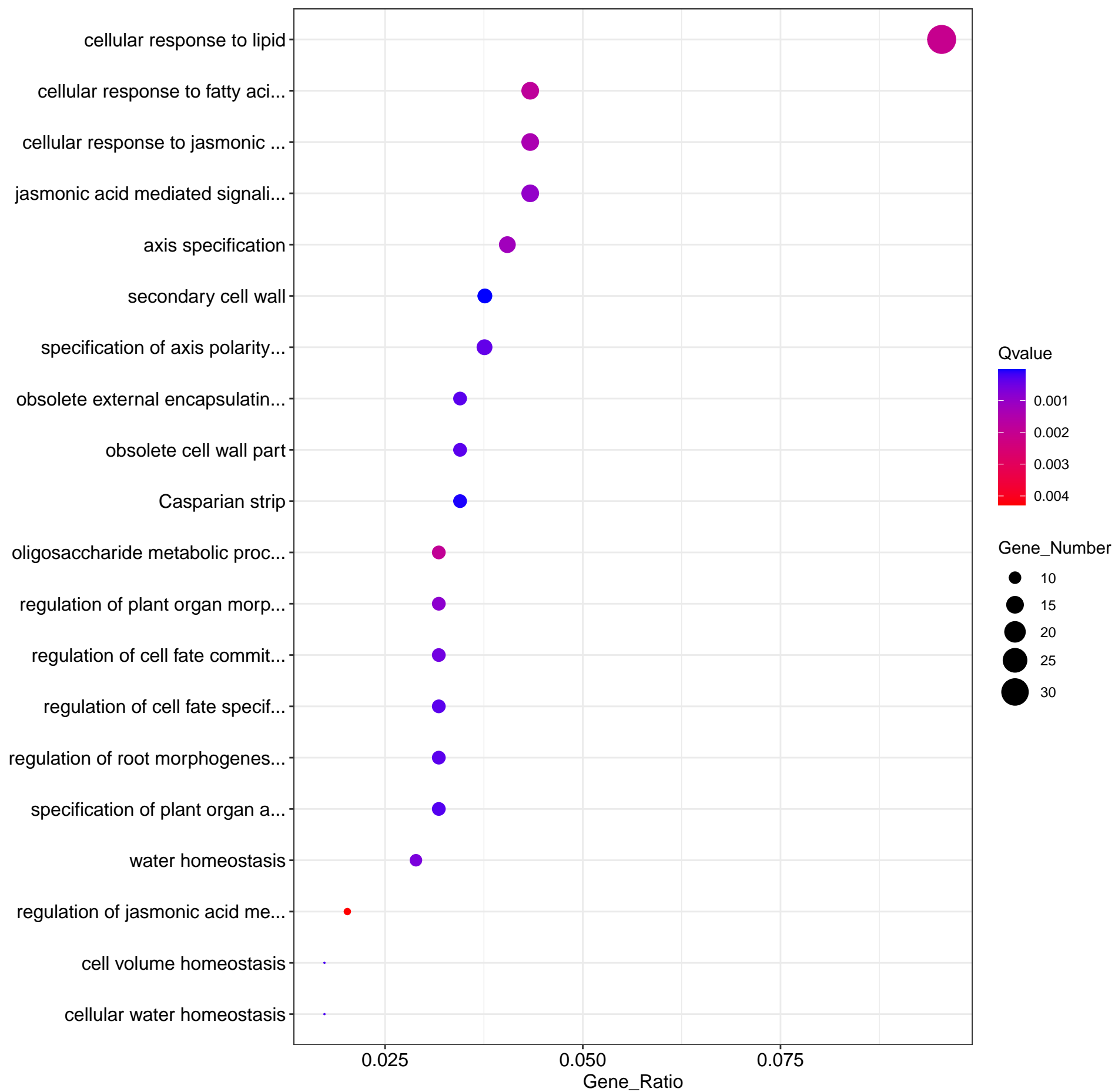

Supplement: Web_Material_uhae252 [file web_material_uhae252.zip › Figure S9.pdf]

turquoise Top 20 GO enrichment result

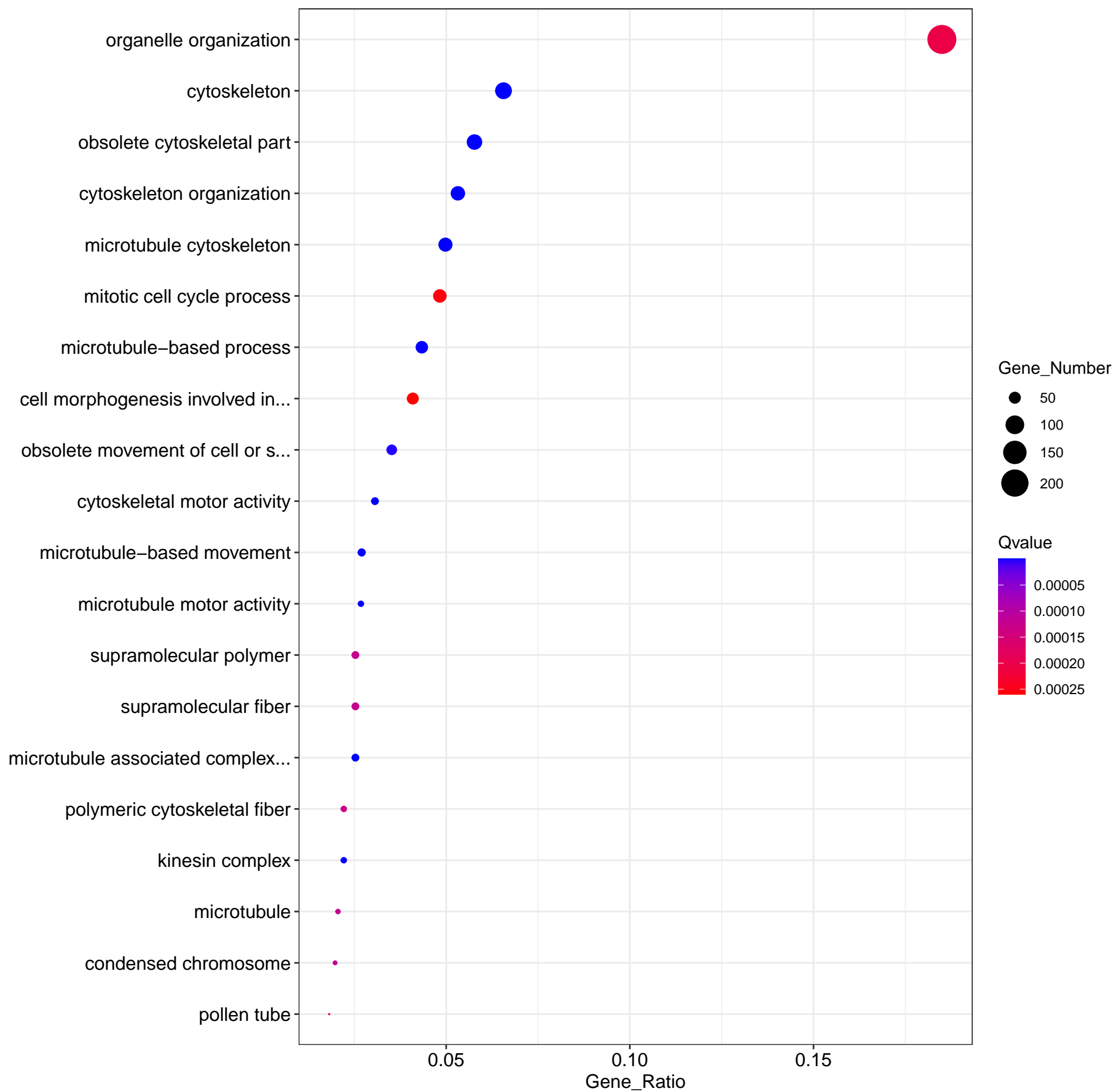

Supplement: Web_Material_uhae252 [file web_material_uhae252.zip › Figure S10.pdf]
